# Network analysis of cold cognition and depression in middle-aged and elder population: the moderation of grandparenting

**DOI:** 10.3389/fpubh.2023.1204977

**Published:** 2023-08-22

**Authors:** Dongling Yuan, Jialing Wu, Shansi Li, Ruoyi Zhang, Xiao Zhou, Yi Zhang

**Affiliations:** ^1^Medical Psychological Center, The Second Xiangya Hospital, Central South University, Changsha, China; ^2^Medical Psychological Institute of Central South University, Central South University, Changsha, China; ^3^National Clinical Research Center on Mental Disorders (Xiangya), Changsha, China

**Keywords:** network analysis, cold cognition, depression, grandparenting, the middle-aged and elder population

## Abstract

**Background:**

Cognitive decline and negative emotions are common in aging, especially decline in cold cognition which often co-occurred with depression in middle-aged and older adults. This study analyzed the interactions between cold cognition and depression in the middle-aged and elder populations using network analysis and explored the effects of grandparenting on the cold cognition-depression network.

**Methods:**

The data of 6,900 individuals (≥ 45 years) from the China Health and Retirement Longitudinal Study (CHARLS) were used. The Minimum Mental State Examination (MMSE) and the Epidemiology Research Center Depression Scale-10 (CESD-10) were used to assess cold cognition and depressive symptoms, respectively. Centrality indices and bridge centrality indices were used to identify central nodes and bridge nodes, respectively.

**Results:**

Network analysis showed that nodes “language ability” and “depressed mood” were more central nodes in the network of cold cognition and depression in all participants. Meantime, nodes “attention,” “language ability” and “hopeless” were three key bridge nodes connecting cold cognition and depressive symptoms. Additionally, the global connectivity of the cold cognition and depression network was stronger in the non-grandparenting than the grandparenting.

**Conclusion:**

The findings shed a light on the complex interactions between cold cognition and depression in the middle-aged and elder populations. Decline in language ability and depressed mood can serve as predictors for the emergence of cold cognitive dysfunction and depression in individuals during aging. Attention, language ability and hopelessness are potential targets for psychosocial interventions. Furthermore, grandparenting is effective in alleviating cold cognitive dysfunction and depression that occur during individual aging.

## Introduction

Aging is increasing seriously globally as the decline in birth rate and increases in life expectancy (1). The statistical data from the National Bureau of Statistics of China show that the number of populations aged 60 and older was 253 million in 2019 (2). Moreover, it is estimated that the number of middle-aged and older adults will reach 400 million in China by 2050, which will bring significant challenges in healthcare and socio-economic (3).

Previous studies have demonstrated that cognitive decline and negative emotions are common in aging, especially decline in cold cognition which often co-occurred with depression in middle-aged and older adults (4, 5). Cold cognition refers to the process of information with less emotional influence, such as working memory, attention, executive function and language (6). Studies have demonstrated that cold cognitive dysfunction is a risk factor for emotional disorders including depression (7, 8). Individuals with cold cognition decline are more vulnerable to suffering from depression than health ones (9). Meanwhile, the meta-analysis has also revealed that some cold cognition dysfunctions are present in the first episode and persist after the depression having been remitted (10–12). These findings suggest that cold cognition dysfunction can be used as a good marker for depression susceptibility (13), prognosis (14) and functional recovery (15). Moreover, a previous study reported that the co-occurrence of cold cognition dysfunction with depression has a negative impact on daily life significantly (16). Although it is well documented that cold cognition impairment and depression often co-exist, they only focused on the relationship between cold cognition and depression at the overall level, ignoring the effects of specific cold cognitive functions on different depressive symptoms. Furthermore, the study on the connection between specific cold cognition dysfunctions and depressive symptoms in middle-aged and older adults is rare.

Recently, increasing numbers of research have found that traditional conceptualization studies of psychopathology obscured meaningful associations between symptoms (17). With the development of cybermetrics, Borsboom (18) has proposed the network theory of psychiatric disorders based on the network analysis approach, which suggests that psychiatric disorders are conceptualized as representations of a symptom network. In the network, nodes are symptoms, the connectivity between nodes represents inter-symptomatic relationships, and the dynamic changes in the symptom network triggered by the interaction of symptoms represent the occurrence and development of psychiatric disorders. Network analysis has emerged as a new approach to investigate complex, dynamic relationships between individual psychiatric symptoms (19). In contrast to traditional perspectives, network analysis treats a particular psychiatric disorder as an interacting cluster of symptoms (18, 20), whereas the emergence and development of disorders are thought to be caused by strong causal interactions between symptoms (18, 21). Thus, an important aim of the network analysis approach is to identify the core symptoms (high centrality nodes) and the bridging symptoms that connect the symptom networks between different psychiatric disorders in the symptom network. Furthermore, core and key bridge symptoms can be more likely to activate other symptoms in the network, thereby driving the development of psychiatric disorders (20, 22). Clinically, core symptoms of psychiatric disorders identified by network analysis can be used as a target of interventions, which may be more effective in preventing the occurrence and promoting regression of psychiatric disorder. Recently, several studies have been conducted to explore psychiatric disorders based on network analysis. For example, Briganti et al. (23) proposed network analysis is a useful tool to explore depression symptoms and offers new insight as to how they interact. A symptom network analysis of PTSD found that fear circuitry and dysphoric PTSD symptoms appear to emerge as connected networks as time elapses after trauma, while intrusive memories and reactivity are centrally associated with other symptoms in the acute phase (22). These findings not only indicate that the relationship between psychiatric symptoms is a network of direct interactions, but also reveal that the clinical symptoms network of psychiatric disorders prominently highlights meaningful associations of symptoms within and between disorders (24). However, network analysis of cold cognition dysfunction and depressive symptoms in middle-aged and older adults is still lack.

In addition, previous studies have also shown that the heterogeneity of depressive symptoms and cold cognitive impairment are largely influenced by individual differences. For example, Sneed and Schulz (25) study in adults found a non-linear decline in cold cognition function with age, while older adults with grandparenting had a slower rate of cognition deterioration than the non-grandparenting. Grandparenting refers to grandparents living with their grandchildren assuming some or all of the responsibility for raising and educating their grandchildren (26), and is very common in China. There is evidence to support the benefits of grandparenting on human well-being and mental health. For example, it was found that older adults who lived with grandchildren had lower levels of depression compared to those without (27). Arpino and Bordone (28) study also found that grandparenting positively affects the cognition function in older adults. Collectively, grandparenting may have important effects on cold cognition and emotion in older adults. However, whether grandparenting plays an important role in cognitive function-depressive networks in middle-aged and older adults needs further confirmation.

In this study, network analysis was used to clarify the complex relationship between cold cognition and depression to identify the core symptoms and the mechanisms of co-occurrence between cold cognition and depression in middle-aged and older adults. Furthermore, we also compared cold cognition-depression networks between the grandparenting and non-grandparenting to explore the effect of grandparenting on the cold cognition-depression network. These may help to explain the negative impacts of cold cognition decline on the emotions of the middle-aged and elder populations, even facilitating timely treatments of core symptoms to prevent and alleviate depression for them.

## Methods

### Participants and procedure

The present study used the China Health and Retirement Longitudinal Survey (CHARLS) in 2018 follow-up data to explore the network structure of cold cognition and depression among middle-aged and older adults in China. The CHARLS is a nationally representative data with a large sample and high data quality regarding basic information, mental health and physical health of Chinese aged 45 years or older.

The inclusion and exclusion criteria for the subjects in this study were (1) having demographic information such as age, gender, whether or not they were raised in intergenerational relationships, and years of education and being older than 45 years old (2); completing the Center for Epidemiologic Studies Depression Scale (CESD-10) and having no missing values; and (3) completing Mini-Mental State Examination (MMSE) assessment with no missing values. There are a total of 19,816 subjects in the 2018 survey. According to the inclusion criteria, 1,107 cases with missing age, gender or age less than 45 years old, and 2,819 cases missing grandparenting status and years of education, 3,109 cases with incomplete CESD scores, and 5,881 cases with incomplete MMSE assessments were excluded, and a final valid sample of 6,900 cases was obtained in present study.

### Measures

#### Cold cognition

The cold cognitive function was calculated by a Minimum Mental State Examination (MMSE) questionnaire in the CHARLS (29). The MMSE scale contains 30 items to cover time and place orientation, immediate memory, attention and calculation, recall, and language ability. The correct answer for each item is recorded as one score. High scores are associated with better cognitive function. While scores below the cut-off values can be considered as an impaired cognition. Specifically, the MMSE cutoff was set at 16/17 for uneducated persons (0 years of education), 19/20 for those with 1–6 years of education, and 23/24 for those with 7 or more years of education (30). The Cronbach’s α coefficient of the MMSE in this study was 0.813.

#### Depression

Center for Epidemiologic Studies Depression-10 (CESD-10) was used to assess depressive symptoms and levels in middle-aged and elder persons (31, 32). The CESD-10 is a self-reported questionnaire to assess depressive symptoms in the past week on a 4-point scale with a total score range of 0 to 30, more severe depression is indicated by a higher score, i.e., the CESD score ≥ 20 was regarded as depressed, ≥ 10 considered to have depressive symptoms and < 10 means no significant depressive symptoms (33). The scale Cronbach’s α coefficient in this study was 0.809.

## Statistical analysis

The data analysis was performed with SPSS and R version 4.1.2.

### Network estimation

The network model of 15 indicators was estimated using the EBIC glasso function in the q graph package of network tools R package (34). Specifically, a Gaussian graphical model was first estimated, which estimated the pairwise correlation parameters among all nodes. Meanwhile, the LASSO regularization technique was employed to cautiously identify correlated edges, and accurately identify the underlying network structure (35). Epskamp and Fried (34) study reported a detailed information on the estimation of such regularized biased correlation networks, and how to estimate such models in R.

For the network with 15 nodes, 105 parameters [15 × (15-1)/2] need to be estimated (34). According to at least 3–5 individuals per parameter, the sample size in this study is sufficient for conducting network analysis (*N* = 6,900). In the network, 15 indicators were depicted as nodes, while the correlations between symptoms were described as edges. The mgm package was used to assess the predictability of each node. A node with a high value of predictability indicated that it is explained by its neighboring nodes (36). Additionally, edges can be positive (green lines) or negative (red lines) and stronger connections were represented by thicker and more saturated edges.

### Centrality and bridge centrality estimation

Three indices of centrality were calculated to assess the importance of each node in the network via the centrality Plot function in the graph package (37) (1). The overall importance of a symptom in the network is indicated by the strength centrality (38) (2); The closeness centrality indicated that the impact of one symptom rapidly spreads to other symptoms (39) (3), The betweenness centrality indicated a bridge symptom connecting with other symptoms and potential target symptoms for interventions (21). Given that the stability of closeness and betweenness centrality are usually low (40), the standardized strength centrality (z scores) was mainly reported in this study, and the results of closeness and betweenness centrality were presented in the Supplementary material.

The network tools R package was used to calculate bridge centrality statistics, including bridge strength, bridge betweenness, and bridge closeness. Similarly, only strength bridge centrality was mainly reported and the top 20% scoring nodes were selected as predicted bridge nodes based on the bridge strength values of the network (41).

### Network accuracy and stability estimation

The accuracy and stability of the network were calculated using bootstrapping methods in the ‘bootnet’ package (34). First, the 95% confidence intervals (CIs) of edge-weights accuracy were computed *via* bootstrapping procedures (bootstrapped samples = 1,000). Secondly, the subset program (subsetting bootstrap; i.e., removing a certain percentage of the participants and re-estimating the network) was used to test the stability of the node centrality estimates. The centrality estimate was considered stable if the centrality order of the network constructed after removing many samples was the same as the centrality order of the original network, the centrality order of the original network was the same as that of the original network. The stability of the centrality indices was then summarized using CS-coefficients (correlation stability). The CS-coefficient should be ideally above 0.5 but at least above 0.25 (42).

### Network comparison test

The comparison of networks between grandparenting and non-grandparenting was calculated using a permutation-based test with the ‘Network Comparison Test’ package (43), which tests differences between two networks based on global strength, network structure, and edge strength. The thresholds to the results of the two networks comparison were adjusted by applying False Discovery Rate (FDR) correction. In addition, centrality estimates were computed using the “test centrality” command, which statistically assesses the centrality of symptoms across the two networks. The magnitude of the node difference was compared using standardized data (z-scores) and evaluated using the effect size (Cohen’s d). Small, medium and high effects are represented by Cohen’s d values of 0.2, 0.4 and 0.8, respectively (44).

## Results

### Participant characteristics

The valid sample size was 6,900 including 3,360 grandparenting (48.70%) and 3,540 non-grandparenting (51.30%), 3,309 males (48.00%) and 3,591 females (52.00%), with the mean age of (63.31 ± 7.07) years and (6.64 ± 3.81) years of education. 1,192 participants did not receive education, 3,024 participants received 1–6 years of education and 2,681 participants received 7 or more years of education; 5,214 participants lived with a spouse (75.57%) and 1,686 participants lived without a spouse (24.43%); 1876 participants were urban residence (27.32%) while 4,991 participants were rural residence (72.68%). Meanwhile, 448 participants had no significant depressive symptoms (6.50%, the score of <10 on the CESD-10), 3,524 participants reported the presence of significant depressive symptoms (51.07%, with a score of ≥10 on the CESD-10). Of these, 2,928 participants were depressed (42.43%, with a score of ≥20 on the CESD-10). 968 paticipants with illiterats showed cognitive impairment (81.21%). 2,205 participants (72.92%) with 1–6 years and 2,360 participants (88.03%) with seven or more years of education exhibited cognition decline, see Appendix 1 for details.

Moreover, Table 1 showed the basic characteristics of the grandparenting and non-grandparenting samples. No significant difference in age, education years and gender between the two groups. The middle-aged and older adults in the grandparenting group had significantly higher levels of cold cognitive function and lower levels of depression than those in the non-grandparenting group. Moreover, scores of five cold cognitive functions (orientation, immediate memory, attention and calculation, recall, language ability) of the grandparenting group were all significantly higher than that of the non-grandparenting group. The grandparenting group also scored significantly lower on all depressive symptoms than the non-grandparenting group. The mean scores, standard deviations, predictability and abbreviation for each node of the MMSE and CESD-10 were presented in Table 2.

**Table 1:** Sample characteristics.

Variables	Total (*n* = 6,900)	Grandparenting (*n* = 3,360)	Non-grandparenting (*n* = 3,540)	*χ^2^/t* (GP *vs* NGP)	*p*
Gender, n (%)	/	/	/	0.44^a^	0.83
Male	3,309 (48.00%)	1,607 (47.82%)	1702 (48.08%)	/	/
Female	3,591 (52.00%)	1753 (52.18%)	1838 (51.92%)	/	/
Age, mean (SD)	63.31 (7.07)	63.46 (6.42)	63.17 (7.63)	1.68^b^	0.09
Education years, mean (SD)	6.64 (3.81)	6.63 (3.69)	6.65 (3.91)	-0.25^b^	0.80
Cognition function, mean (SD)	14.16 (6.71)	15.21 (6.73)	13.17 (6.53)	12.73 ^b^	<0.001
Depression, mean (SD)	19.19 (6.51)	17.71 (5.63)	20.60 (6.9)	−19.03 ^b^	<0.001

Total, total sample; GP, Grandparenting sample; NGP, Non-grandparenting sample; a, the test statistic from the Chi-Squared test; b, the test statistic from the independent samples *t*-test.

**Table 2:** Each node of the MMSE and CESD-10 in total, grandparenting and non-grandparenting sample.

Nodes	Abbreviation	*M* (SD)			Pre		
		Total	GP	NGP	Total	GP	NGP
Cognitive functions (The MMSE)							
Orientation	Orientation	6.12 (2.65)	6.13 (2.69)	5.77 (2.55)	0.05	0.03	0.08
Memory	Memory	1.25 (1.34)	1.45 (1.37)	1.16 (1.31)	0.72	0.74	0.69
Attention	Attention	2.26 (2.30)	2.47 (1.96)	2.12 (1.96)	0.10	0.07	0.12
Recall	Recall	2.02 (1.04)	1.22 (1.29)	0.97 (1.22)	0.61	0.62	0.58
Language ability	Language ability	3.67 (3.04)	3.93 (3.07)	3.16 (2.73)	0.72	0.75	0.68
Depressive symptoms (CESD-10)							
I was bothered by things that usually do not bother me.	Upset	1.96 (1.09)	1.79 (1.00)	2.16 (1.13)	0.38	0.31	0.41
I had trouble keeping my mind on what I was doing.	Mind distraction	1.97 (1.08)	1.85 (1.01)	2.13 (1.12)	0.35	0.30	0.37
I felt depressed.	Depressed mood	1.96 (1.08)	1.79 (0.98)	2.14 (1.37)	0.48	0.44	0.49
I felt that everything I did was an effort.	Exhaust	2.03 (1.15)	1.87 (1.07)	2.20 (1.19)	0.38	0.32	0.41
I felt hopeful about the future.	Hopeless	2.35 (1.23)	2.20 (1.18)	2.38 (1.22)	0.16	0.18	0.14
I felt fearful.	Fear	1.42 (0.84)	1.29 (0.67)	1.51 (0.93)	0.24	0.17	0.27
My sleep was restless.	Sleeplessness	2.18 (1.22)	2.07 (1.18)	2.35 (1.24)	0.18	0.13	0.21
I was happy.	Unhappy	2.09 (1.18)	1.97 (1.14)	2.22 (1.20)	0.25	0.22	0.27
I felt lonely.	Lonely	1.69 (1.05)	1.50 (0.90)	1.86 (1.14)	0.32	0.25	0.34
I could not “get going”.	Cannot continue	1.49 (0.92)	1.34 (0.76)	1.60 (1.01)	0.35	0.28	0.38

M, mean; SD, Standard deviation; Pre, Predictability; Total, total sample; GP, Grandparenting sample; NGP, Non-grandparenting sample.

### Network estimation for total samples

#### Network structure

**Figure 1:**
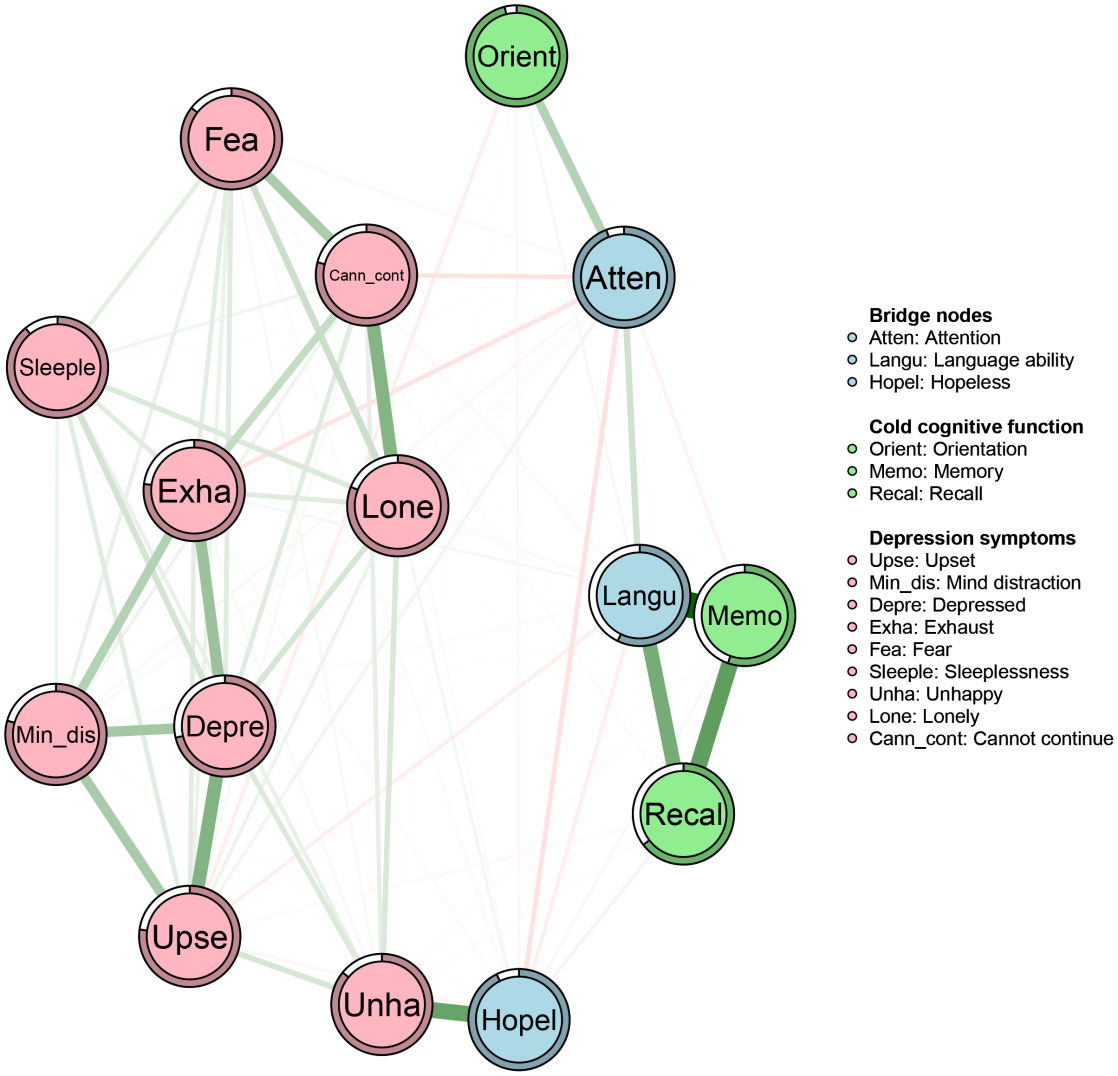
Networks of cold cognition and depressive symptoms in middle-aged and elder population. Nodes represent cold cognition and depressive symptoms and edges represent partial correlations between symptoms. Edge thickness and darkness indicate the association strength (minimum and maximum edge values were set to be equal across networks), and edge color indicates the correlation valence (green = positive; red = negative). Bridge nodes indicated the connection of cold cognition and depressive symptoms. Orient, Orientation; Memo, Memory; Atten, Attention; Recal, Recall; Langu, Language ability; Upse, Upset; Min_dis, Mind distraction; Depre, Depressed mood; Exha, Exhaust; Hopel, Hopeless; Fea, Fear; Sleeple, Sleeplessness; Unha, Unhappy; Lone, Lonely; Cann_cont, Cannot continue.

The network structure of cold cognition and depression in middle-aged and older adults was depicted in Figure 1. The ring-shaped pie charts of the network were used to indicate the predictability of nodes, i.e., the mean predictability was 0.35 (range from 0.05 to 0.72, Table 2). Within the cold cognition community, node “memory” had the most directly positive connection with node “language ability,” followed by the positive connection between nodes “memory” and “recall.” Moreover, “memory,” “recall” and “language ability” were more closely linked to each other in the cold cognitive function cluster. Among the depressive symptom community, node “unhappy” had the most directly positive with node “hopeless,” followed by the positive connection between nodes “lonely” and “cannot continue.” Furthermore, “upset,” “mind distraction,” “depressed mood” and “exhaust” were more closely linked to each other, followed by the degree of the closeness among “fear,” “lonely” and “cannot continue.” Additionally, there were many associations among the items across the communities. For example, node “hopeless” was most strongly negatively associated with node “attention,” followed by connections between nodes “attention,” “exhaust,” and “cannot continue.” Simultaneously, not all nodes in the two sub-networks of cold cognition and depression were directly connected, e.g., nodes “lonely” and “depressed mood” were not significantly directly connected to the cluster of cold cognition nodes.

#### Centrality and bridge centrality

**Figure 2:**
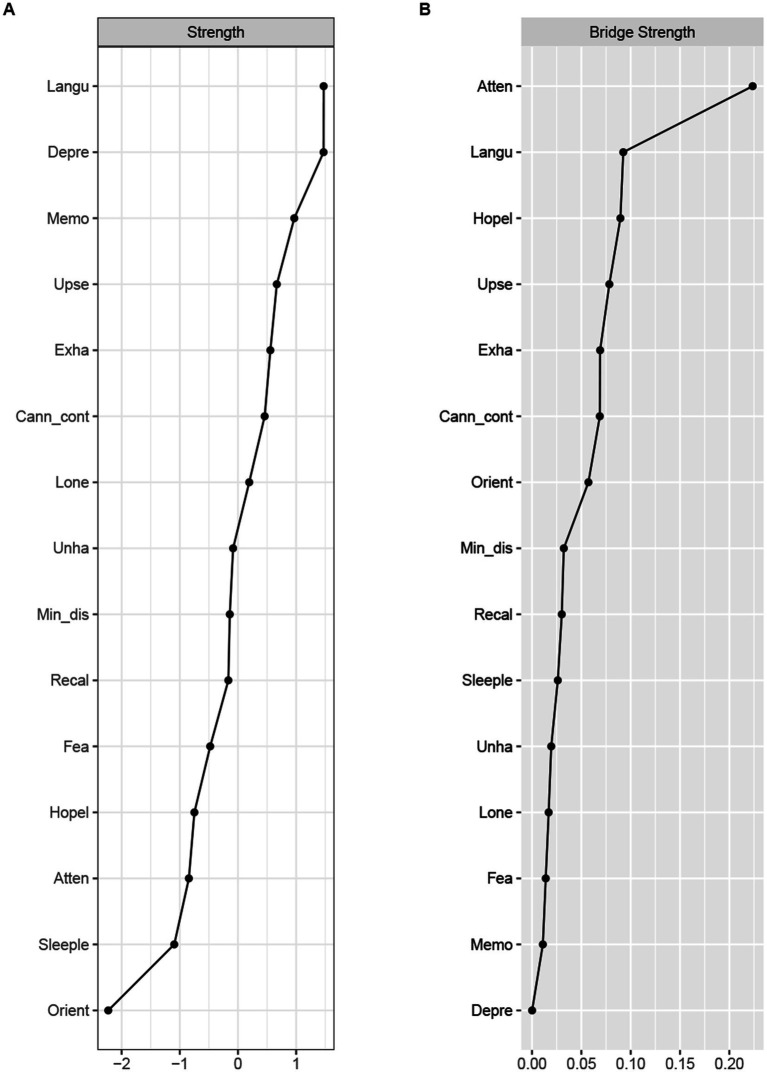
Node strength **(A)** and bridge strength **(B)** centrality estimates for the cold cognition and depressive symptoms network in middle-aged and elder population. Standardized z-scores are plotted for ease of interpretation. Higher scores represent higher centrality estimates (i.e., the symptom has greater influence in the network). Orient, Orientation; Memo, Memory; Atten, Attention; Recal, Recall; Langu, Language ability; Upse, Upset; Min_dis, Mind distraction; Depre, Depressed mood; Exha, Exhaust; Hopel, Hopeless; Fea, Fear; Sleeple, Sleeplessness; Unha, Unhappy; Lone, Lonely; Cann_cont, Cannot continue.

The centrality and bridge strength of each node among middle-aged and older adults were presented in Figure 2 (results of other centrality indicators and the estimation of node strength difference are in Appendix S2). Node “language ability” had the highest strength. Nodes “depressed mood,” “memory,” and “upset” were also statistically stronger than most other nodes in the network. The bridge strength in nodes “attention,” “language ability” and “hopeless” were stronger than most other nodes.

#### Network accuracy and stability

The edge stability estimation showed moderate stability of the estimated networks: although there were considerable overlaps among the 95% CIs of edge weights, nonoverlapped CIs also existed (Appendix S3) and estimation of edge weight difference indicated edges of higher stability were significantly different with other edges in the network (Appendix S4). Meanwhile, stability estimates of the centrality index showed that the centrality strength stability coefficient (CS-coefficients) was 0.750 (Appendix S5).

### Cold cognition-depression networks in grandparenting and non-grandparenting

#### Network structure

**Figure 3:**
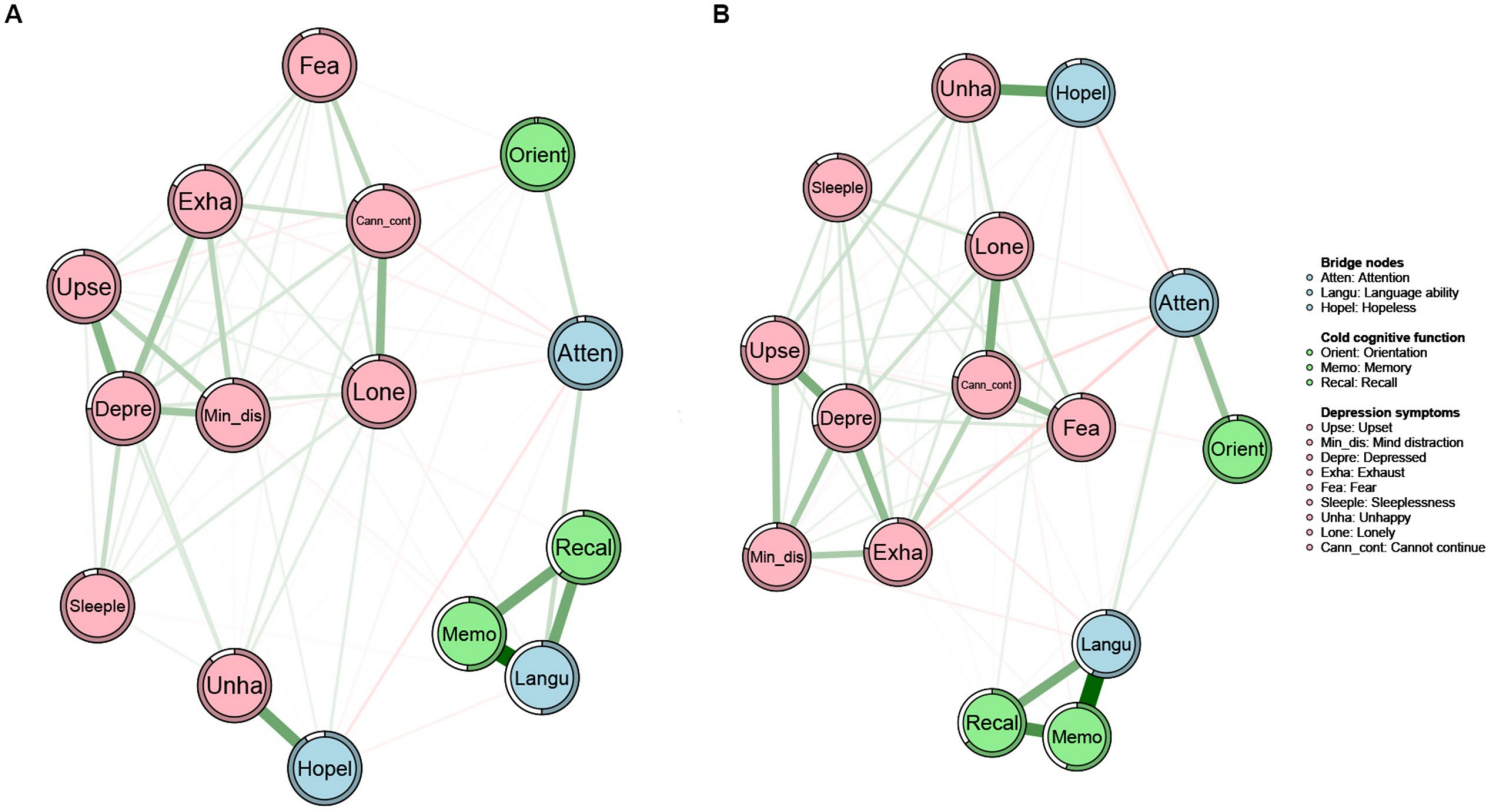
Networks of cold cognition and depressive symptoms in the grandparenting **(A)** and non-grandparenting **(B)**. Nodes represent cold cognition and depressive symptoms and edges represent partial correlations between symptoms. Edge thickness and darkness indicate the association strength (minimum and maximum edge values were set to be equal across networks), and edge color indicates the correlation valence (green = positive; red = negative). Bridge nodes indicated the connection of cold cognition and depressive symptoms. Orient, Orientation; Memo, Memory; Atten, Attention; Recal, Recall; Langu, Language ability; Upse, Upset; Min_dis, Mind distraction; Depre, Depressed mood; Exha, Exhaust; Hopel, Hopeless; Fea, Fear; Sleeple, Sleeplessness; Unha, Unhappy; Lone, Lonely; Cann_cont, Cannot continue.

The network structure of cold cognition and depression among middle-aged and older adults with and without grandparenting was depicted in Figure 3. Although the overall structure is similar, there were significant differences in the pattern of nodes’ connections and the strength of the edges between the two networks. Additionally, the ring-shaped pie charts of the grandparenting and non-grandparenting networks were used to indicate the predictability of nodes, i.e., the mean predictability was 0.32 (range from 0.03 to 0.75) and 0.36 (range from 0.08 to 0.69), respectively (Table 2).

#### Centrality and bridge centrality

**Figure 4:**
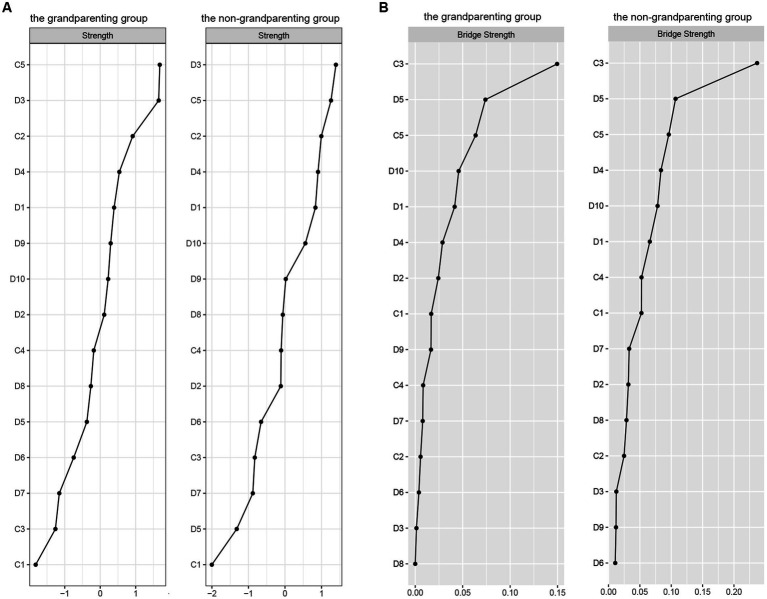
Node strength **(A)** and bridge strength **(B)** centrality estimates for the cold cognition and depressive symptoms network in the grandparenting and non-grandparenting. Standardized z-scores are plotted for ease of interpretation. Higher scores represent higher centrality estimates (i.e., the symptom has greater influence in the network). Orient, Orientation; Memo, Memory; Atten, Attention; Recal, Recall; Langu, Language ability; Upse, Upset; Min_dis, Mind distraction; Depre, Depressed mood; Exha, Exhaust; Hopel, Hopeless; Fea, Fear; Sleeple, Sleeplessness; Unha, Unhappy; Lone, Lonely; Cann_cont, Cannot continue.

The centrality and bridge strength of each node in grandparenting and non-grandparenting networks were shown in Figure 4 (results of other centrality indicators and the estimation of node strength difference were presented in Appendix S6). Node “language ability” had the highest strength, while nodes “depressed mood,” “memory,” and “upset” were also statistically stronger than most other nodes in the grandparenting network. In the non-grandparenting network, the strength of node “depressed mood” was highest, followed by nodes “language ability,” “memory,” and “upset.” In addition, nodes “attention,” “hopeless” and “language ability” were stronger than most other nodes for the bridge strength of the two networks, which is consistent with the bridge nodes of the cold cognition and depression network in the total sample.

#### Network accuracy and stability

The edge stability estimation showed moderate stability of the grandparenting and non-grandparenting networks (Appendix S7) and the estimation of edge weight difference showed that edges of higher stability of these two networks were significantly different from other edges (Appendix S8). Meanwhile, stability estimates of the centrality index showed that the centrality strength stability coefficients (CS-coefficients) were 0.75 in the two networks (Appendix S9).

#### Network comparison

The results of the network comparison showed that the grandparenting and non-grandparenting networks differed significantly in overall structure (*M* = 0.09, *p* = 0.02) and connectivity (*S* = 0.61, *p* < 0.01). The non-grandparenting network had higher global connectivity than the grandparenting network (6.03 vs. 5.41). The comparison of mean node strength centrality was presented in Table 3. The nodes in the non-grandparenting network had significantly higher centrality indices than nodes in other networks on “orientation,” “attention,” “recall,” “upset,” “exhaust,” “sleeplessness,” “unhappy,” “lonely” and “cannot continue” with Cohen’s d values above 0.8 (Table 3).

**Table 3:** Comparison of mean node strength centrality between the grandparenting and non-grandparenting.

	*M* (SD)			
Nodes	Grandparenting	Non-grandparenting	*p*	Cohen’s d
Orientation	0.225 (0.053)	0.362 (0.031)	0.009	−3.155
Memory	0.989 (0.032)	1.020 (0.037)	0.336	/
Attention	0.392 (0.043)	0.545 (0.051)	0.009	−3.243
Recall	0.721 (0.030)	0.802 (0.038)	0.019	−2.366
Language ability	1.120 (0.035)	1.070 (0.035)	0.138	/
Upset	0.808 (0.043)	0.956 (0.043)	0.009	−3.441
Mind distraction	0.760 (0.038)	0.823 (0.031)	0.168	/
Depressed mood	1.200 (0.038)	1.150 (0.031)	0.267	/
Exhaust	0.893 (0.041)	0.982 (0.036)	0.039	−2.306
Hopeless	0.563 (0.050)	0.510 (0.049)	0.475	/
Fear	0.579 (0.038)	0.707 (0.040)	0.019	−0.579
Sleeplessness	0.470 (0.035)	0.623 (0.030)	0.009	−4.693
Unhappy	0.707 (0.036)	0.819 (0.031)	0.009	−3.334
Lonely	0.813 (0.043)	0.854 (0.034)	0.445	/
Cannot continue	0.841 (0.038)	0.960 (0.034)	0.009	−3.300

## Discussion

The present study used network analysis to construct the network of cold cognition and depression in middle-aged and elder adults. The key findings are that (1): the cold cognitive cluster and depressive symptoms cluster are relatively independent in the middle-aged and elder adults (2); language ability and depressed mood are the most central nodes in the cold cognitive cluster and depressive symptoms cluster, respectively, as well as in the cold cognition-depression network; while attention, language ability, and hopeless are the top-three key bridge nodes connecting cold cognition and depressive symptoms in the network (3); There are significant effects of grandparenting on the global structure and connectivity of the network, specifically, the connectivity of the cold cognition-depression network is significantly stronger in the non-grandparenting than that in the grandparenting; the non-grandparenting network nodes have higher centrality indices on orientation, attention, recall, upset, exhaust, sleeplessness, unhappy and lonely.

Brain organs gradually degenerate with aging, which leads to the decline of cognitive function, especially cold cognitive functions such as attention, working memory, and language ability. In this study, the language ability, assessed by the MMSE, is a comprehensive cold cognitive ability. Depressed mood, as a core symptom of depression (45), covers a series of negative emotional experiences, such as sadness and low mood (46). Due to the gradual decline of physical functions (including brain functions) and faded social roles, middle-aged and elder persons are prone to develop a depressed mood, even leading to depressive episodes (47). In this study, language ability is the core node in both the cold cognitive clusters and the cold cognition-depression network, while the cold cognitive ability shows an overall decline in middle-aged and elder population. Consistent with previous reports (36, 48), the depressed mood is another core symptom in both the depressive symptom cluster and cold cognition-depression network in middle-aged and older adults. In consideration of the vital roles of the highest centrality nodes in a network (48, 49), the language ability decline and depressed mood are the most likely candidates for triggering and maintaining the decline in cold cognition and depressive symptoms. According to the network theory of mental disorder (18), targeting central symptoms with biological or psychosocial interventions, rather than peripheral symptoms, can be highly effective. In present study, interventions for the two core symptoms (depressed mood and language skills) would alleviate depressed mood and slow down the decline of language skills in middle-aged and older adults, as well as facilitate the improvement of peripheral symptoms, such as decreased attention and hopelessness, which in turn would have a greater impact on the cold cognitive-depression network (20). However, we also found the lack of a significantly direct connection between depressed mood and cold cognition node clusters, which not only supports the hot and cold cognition model (50), but also hints that reducing core symptoms, such as depressed mood or enhancing language ability, alone only improves the internal symptom network of depression or cold cognition, respectively.

According to the network theory of psychiatric disorders (18), finding and targeting key bridge-connecting symptoms (nodes) is needed to treat with cold cognitive-depressive co-morbidities in middle-aged and older adults. In the present study, attention, language ability and hopelessness are the top-three key bridge nodes connecting cold cognition to depressive symptoms in the network, suggesting that bridge nodes contribute to comorbidity of cold cognitive dysfunction and depression. In clinically, targeting the three bridge nodes of attention, language skills, and hopelessness for interventions may be effective for both cold cognitive dysfunction and depressive symptoms (51). The hopelessness theory of depression (52) proposes that adverse life events are prone to induce despair, helplessness, and uselessness in individuals, resulting in depressed mood and thoughts. In this study, hopelessness is a stronger symptom linking cold cognition and depressive symptoms. In middle-aged and elder individuals, cold cognitions (such as working memory and attention) are markedly declined, and their social roles are gradually becoming less important until retirement, which may be a series of adverse stressors and lead to senses of uselessness and hopelessness, and in turn hopelessness trigger the most core symptoms of depression - depressed mood and eventually develop into depressive disorder. Thus, hopelessness in middle-aged and older adults is not only a symptom of depression but may also be a risk factor for developing into depressive disorder. The findings of this study combined with previous studies may have utility in tailoring and testing interventions to reduce the co-occurrence of cold cognitive dysfunctions and depression in the mild-aged and elder population. For example, it is recommended to maintain healthy lifestyle to slow down physiological aging (including cold cognition), while communication with good friends and family members is thought to alleviate negative mood symptoms (53).

In China, middle-aged and older adults often regard parenting their grandchildren as a responsibility and obligation. Previous studies suggested that intergenerational parenting may be a protective factor in cognitive impairment and depression (27, 28). This study also showed that the global connectivity of the cold cognition-depression network in the non-grandparenting is stronger than that in the grandparenting. Also, grandparenting failed to change the effect of cold cognition on depressive symptoms but had a moderating effect on the strength of the connection in the cold cognition – depressive symptom network. In addition, non-grandparenting group is more strongly connected to this network, which suggested that it is at higher risk for depression as cold cognitive functioning declines in mild-aged and elder adults without grandparenting.

Borsboom et al. (20) believed that high centrality symptoms increasingly activate other symptoms in the network and improve the development of psychiatric disorders. In this study, three-fifths of centrality nodes in the cold cognitive-depressive symptom network were much higher in the non-grandparenting group than in the grandparenting group, suggesting a protective effect of grandparenting on comorbidity of cold cognitive dysfunction and depression. Additionally, non-emotional cold cognitive impairment contributes to the development of hot cognition, and subsequently lead to psychiatric symptoms and further worsening cold cognitive impairment (12). Specifically, cold cognition decline such as attention and recall are prone to activate other emotional and somatic symptoms in the network among middle-aged and older adults without grandparenting, which would promote the development of co-occurrence in cold cognitive disorder and depression.

Finally, the present study also has some limitations: (1) although the effect of cold cognition on depression has received increasing attention from clinical studies, the role of hot cognition on depression should not be neglected. (2) The present study explored the relationship between cold cognition and depressive symptoms from a perspective network analysis, which has some clinical implications for improving cold cognition impairment and depression in the middle-aged and older adults. However, future studies should analyze their dynamic relationships based on longitudinal data to guide clinical practice with more robust and rigorous findings; (3) although the MMSE is a broad measure of cognitive ability and cold cognition is a complex ability, more precise and comprehensive neurofunctional tests are needed in the future to assess cold cognition and depressive network structure in middle-aged and older adults; (4) excepting for grandparenting, future research also needs to study the effects of other factors, such as socioeconomic status, physical and mental health status, on the association between cold cognitive and depressive symptoms in middle-aged and older adults.

In conclusion, there are complex interactions between cold cognition and depression in middle-aged and older adults. Grandparenting has effect on the cold cognitive-depression networks. Systemic multi-level interventions targeting central nodes and bridge nodes may be effective in alleviating the co-occurrence of cold cognitive cline and depressive symptoms in the mild-aged and older adult population.

## Data availability statement

The original contributions presented in the study are included in the article/Supplementary material, further inquiries can be directed to the corresponding author.

## Ethics statement

Ethical review and approval was not required for the study on human participants in accordance with the local legislation and institutional requirements. Written informed consent for participation was not required for this study in accordance with the national legislation and the institutional requirements.

## Author contributions

YZ and DY: conception and design. DY, JW, and SL: provision of study materials. DY, JW, RZ, and XZ: collection and assembly of data. DY and YZ: data analysis, interpretation, and manuscript writing. All authors contributed to the article and approved the submitted version.

## Funding

This work was supported by Hunan Provincial Natural Science Foundation China (No. 2022JJ70146), the Hunan Provincial Innovation Foundation for Postgraduate (No. CX20230299) and the Fundamental Research Funds for the Central Universities of Central South University (No. 2023ZZTS0224).

## Conflict of interest

The authors declare that the research was conducted in the absence of any commercial or financial relationships that could be construed as a potential conflict of interest.

## Publisher’s note

All claims expressed in this article are solely those of the authors and do not necessarily represent those of their affiliated organizations, or those of the publisher, the editors and the reviewers. Any product that may be evaluated in this article, or claim that may be made by its manufacturer, is not guaranteed or endorsed by the publisher.
