# Supplementary material for: Network analysis of cold cognition and depression in middle-aged and elder population: the moderation of grandparenting
Source: Front Public Health. 2023 Aug 22;11:1204977. doi: 10.3389/fpubh.2023.1204977 (PMC10479032; doi:10.3389/fpubh.2023.1204977)
Supplement: Supplementary file 1 [file Data_Sheet_1.pdf]

Supplementary Material

Network analysis of cold cognition and depressive symptoms in middle-aged and elder population: The moderation of grandparenting

Dongling Yuan[1], Jialing Wu[1], Shansi Li[1], Ruoyi Zhang, Xiao Zhou, Yi Zhang[1,2,3*]

***Correspondence:** Yi Zhang; Email: zhangyipsy@csu.edu.cn

Network estimation for total samples

Appendix S1-1 Node strength centrality estimates for the cold cognition and depressive symptoms network in total samples. Standardized z-scores are plotted for ease of interpretation. Higher scores represent higher centrality estimates (i.e. the symptom has greater influence in the network). Orient, "Orientation"; Memo, "Memory"; Atten, "Attention"; Recal, "Recall"; Langu, "Language ability"; Upse, "Upset"; Min_dis, "Mind distraction"; Depre, "Depressed mood"; Exha, "Exhaust"; Hopel, "Hopeless"; Fea, "Fear"; Sleeple, "Sleeplessness"; Unha, "Unhappy"; Lone, "Lonely"; Cann_cont, "Cannot continue", same as below.

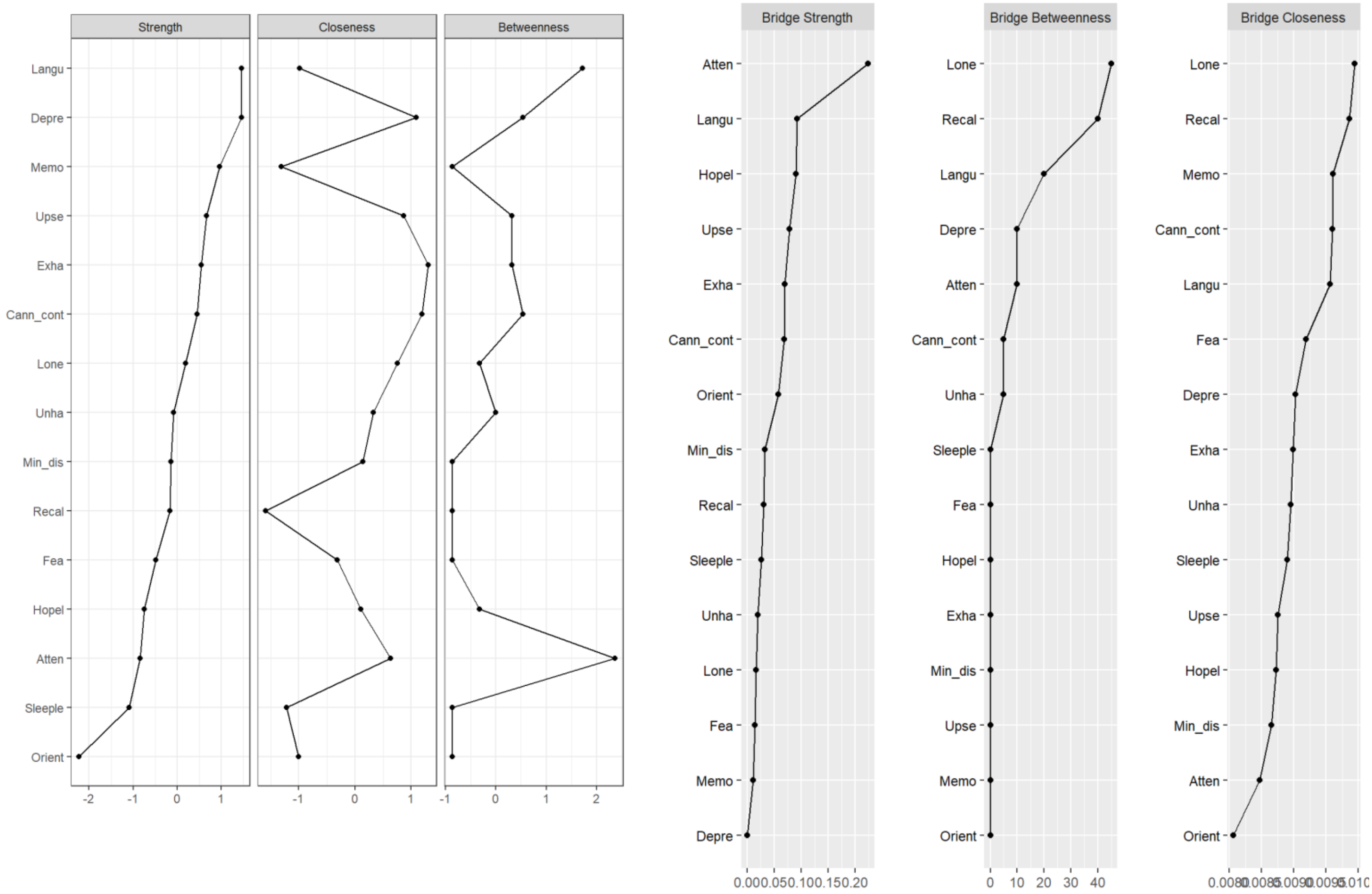

Appendix S1-2 Estimation of node strength difference by bootstrapped difference test. Bootstrapped difference tests between node strength of factors. Gray boxes indicate nodes that do not significantly differ from one-another. Black boxes represent nodes that significantly differ from one another (α = 0.05). White boxes show the values of node strength.

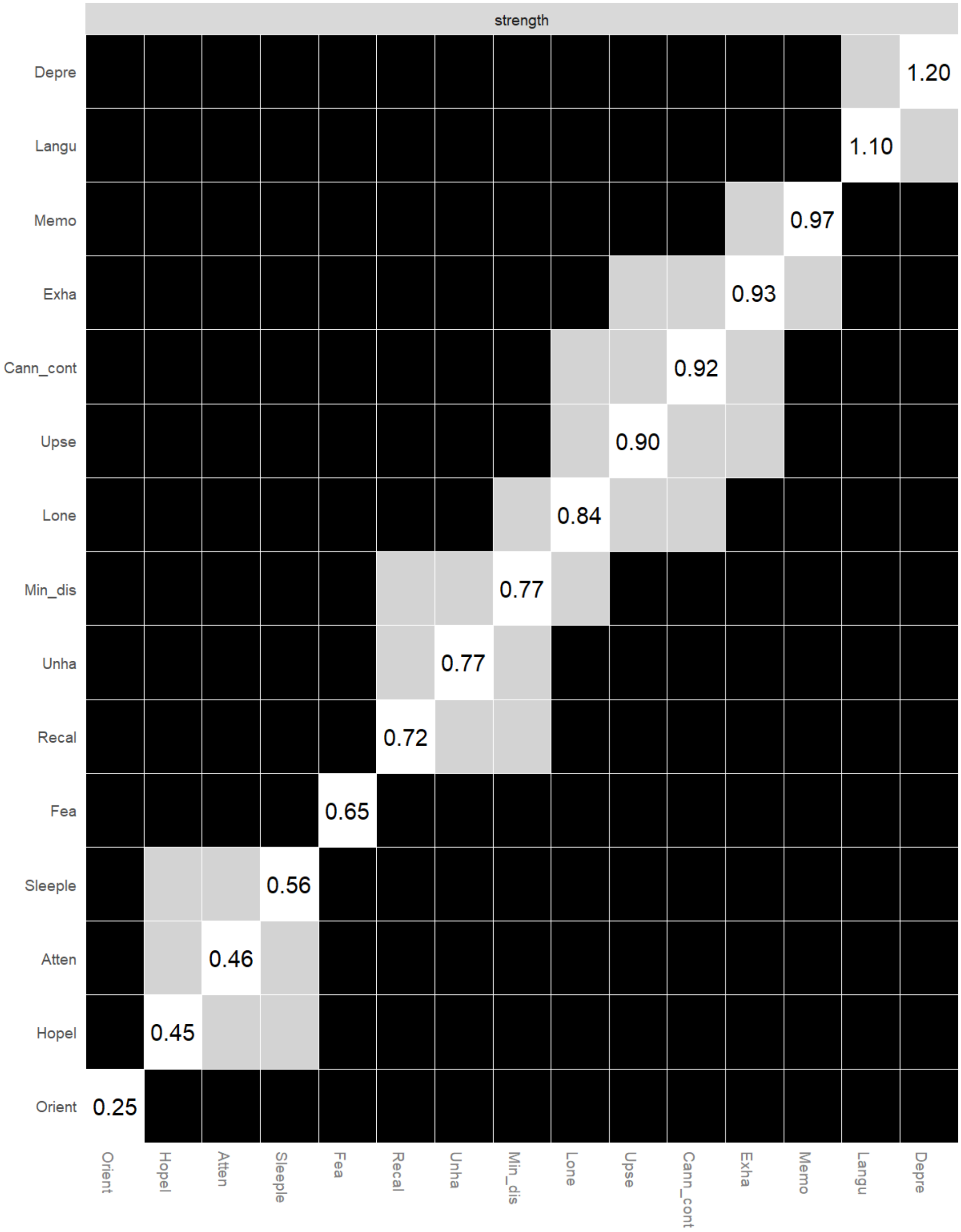

Appendix S2

Accuracy of the edge-weights for the current network model. The gray area represents the 95% Confidence Intervals of edge weights, estimated with the non-parametric bootstrap procedure. Wide intervals indicate lower stability and narrow intervals indicate higher stability. The red dots indicate the sample values, while the black dots indicate the values of each edge weight, ordered from the highest to the lowest value

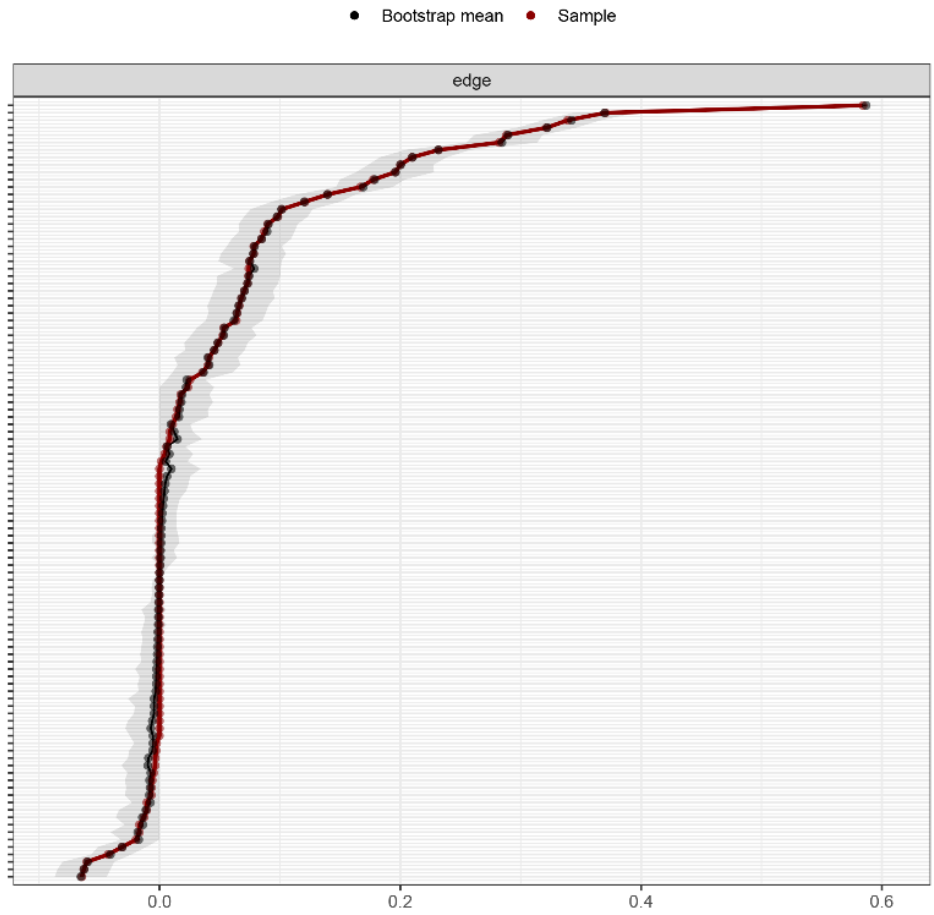

Appendix S3 Estimation of edge weight difference by bootstrapped difference test. Bootstrapped difference tests between edge weights in the network. Gray boxes indicate edges that do not significantly differ from one another. Black boxes represent edges with significant difference from one another (α = 0.05). Blue boxes in the edge-weight plot indicate positive correlations, and orange boxes in the edge-weight plot indicate negative correlations.

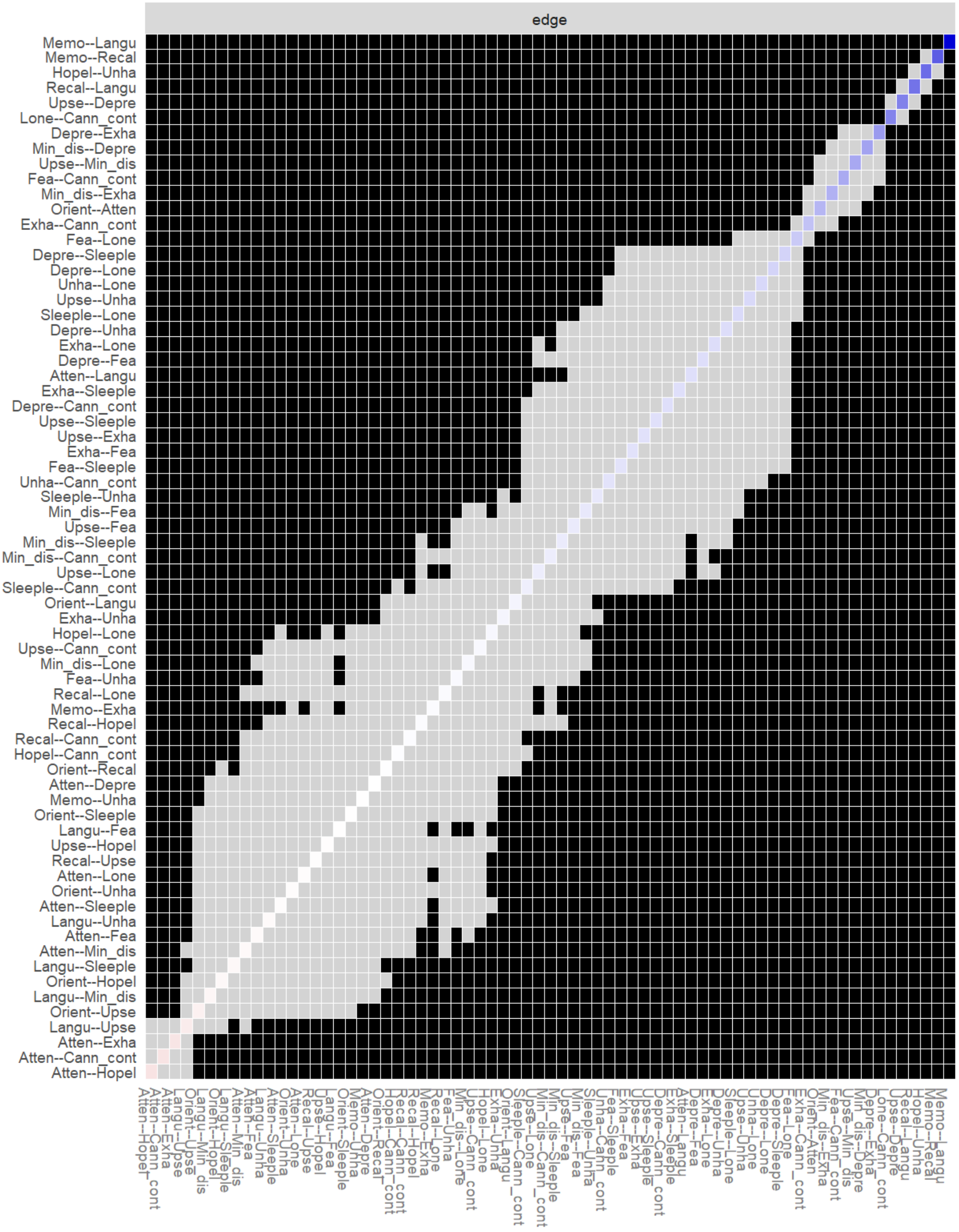

Appendix S4 Stability of centrality indices by case dropping subset bootstrap. The x-axis represents the percentage of cases of original sample used at each step. The y axis represents the average of correlations between the centrality indices from the original network and the centrality indices from the networks that were re-estimated after dropping increasing percentages of cases. Each line indicates the correlations of betweenness, strength and closeness, while areas indicate 95% CI.

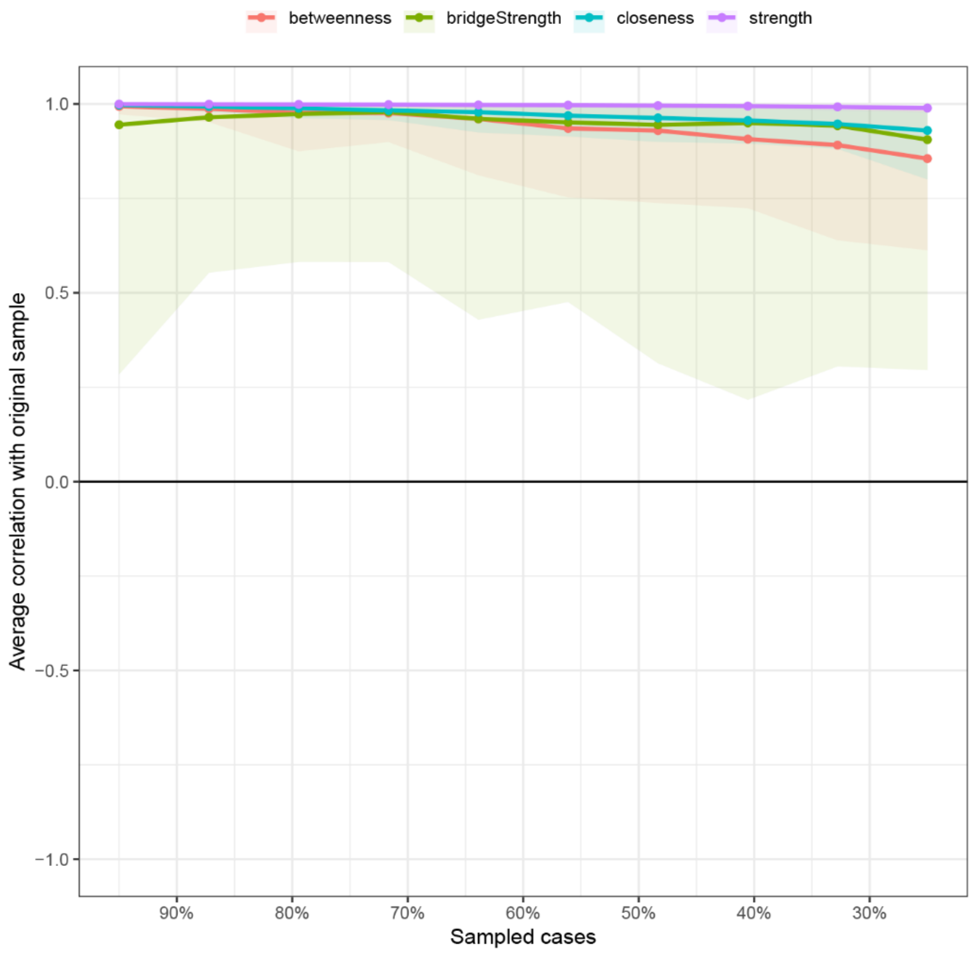

Maximum drop proportions to retain correlation of 0.7 in at least 95% of the samples:

betweenness: 0.283

- For more accuracy, run bootnet(..., caseMin = 0.206, caseMax = 0.361)

bridgeStrength: 0.361

- For more accuracy, run bootnet(..., caseMin = 0.283, caseMax = 0.439)

closeness: 0.75 (CS-coefficient is highest level tested)

- For more accuracy, run bootnet(..., caseMin = 0.672, caseMax = 1)

strength: 0.75 (CS-coefficient is highest level tested)

- For more accuracy, run bootnet(..., caseMin = 0.672, caseMax = 1)

Cold cognition-depression networks in grandparenting and non-grandparenting

Appendix S5-1 Node strength centrality estimates for the cold cognition and depressive symptoms network in grandparenting and non-grandparenting. Standardized z-scores are plotted for ease of interpretation. Higher scores represent higher centrality estimates (i.e. the symptom has greater influence in the network). Orient, "Orientation"; Memo, "Memory"; Atten, "Attention"; Recal, "Recall"; Langu, "Language ability"; Upse, "Upset"; Min_dis, "Mind distraction"; Depre, "Depressed mood"; Exha, "Exhaust"; Hopel, "Hopeless"; Fea, "Fear"; Sleeple, "Sleeplessness"; Unha, "Unhappy"; Lone, "Lonely"; Cann_cont, "Cannot continue", same as below.

Grandparenting

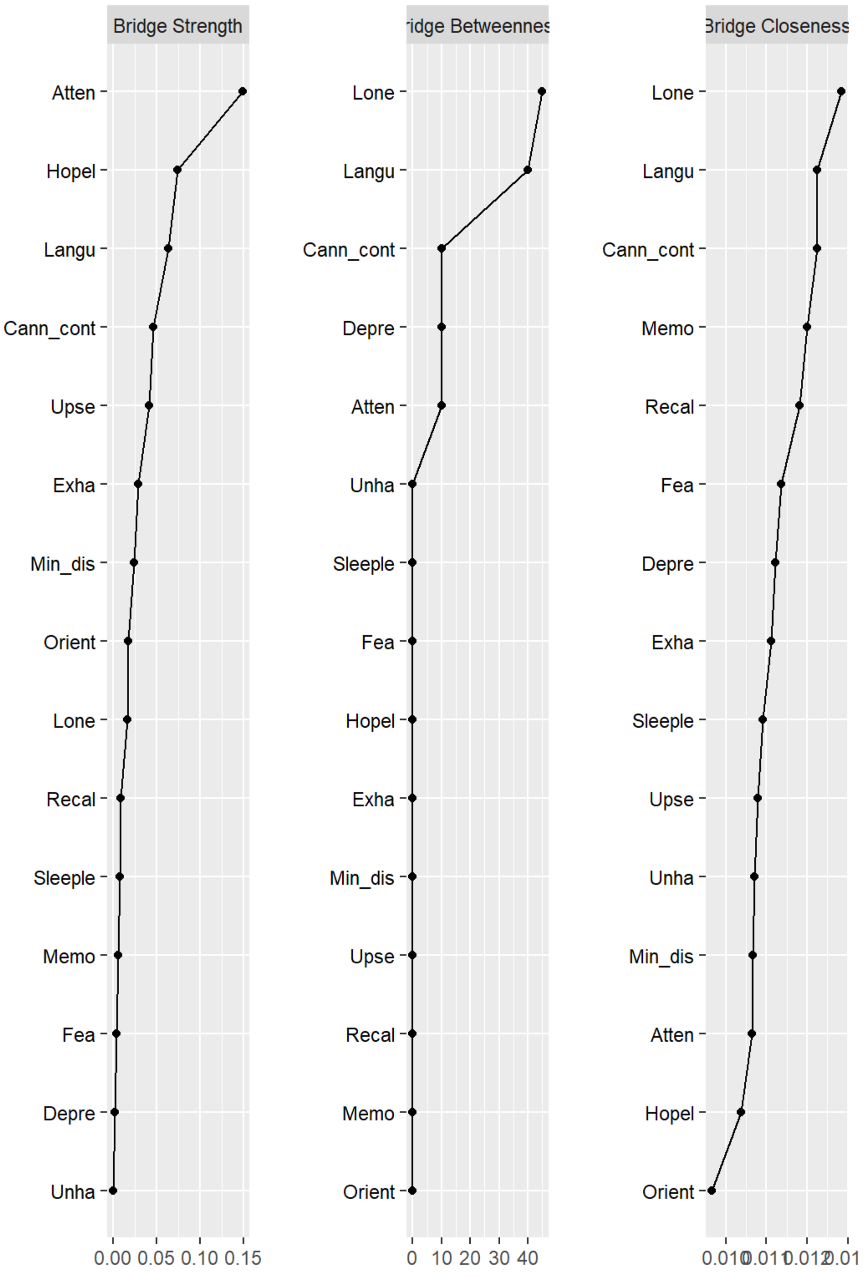

Non-grandparenting

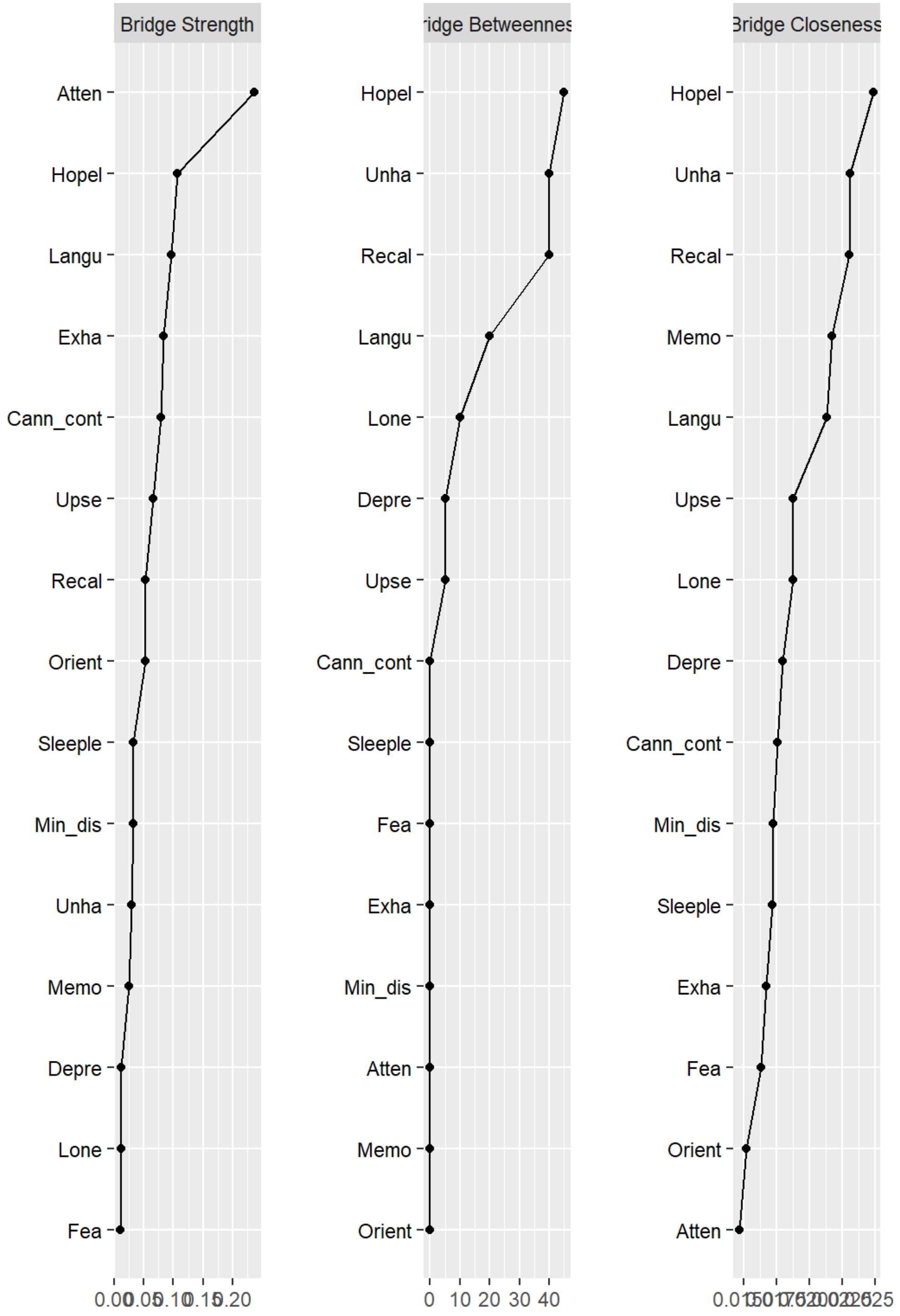

Figure S5-2 Estimation of node strength difference by bootstrapped difference test. Bootstrapped difference tests between node strength of factors. Gray boxes indicate nodes that do not significantly differ from one-another. Black boxes represent nodes that significantly differ from one another (α = 0.05). White boxes show the values of node strength.

Grandparenting

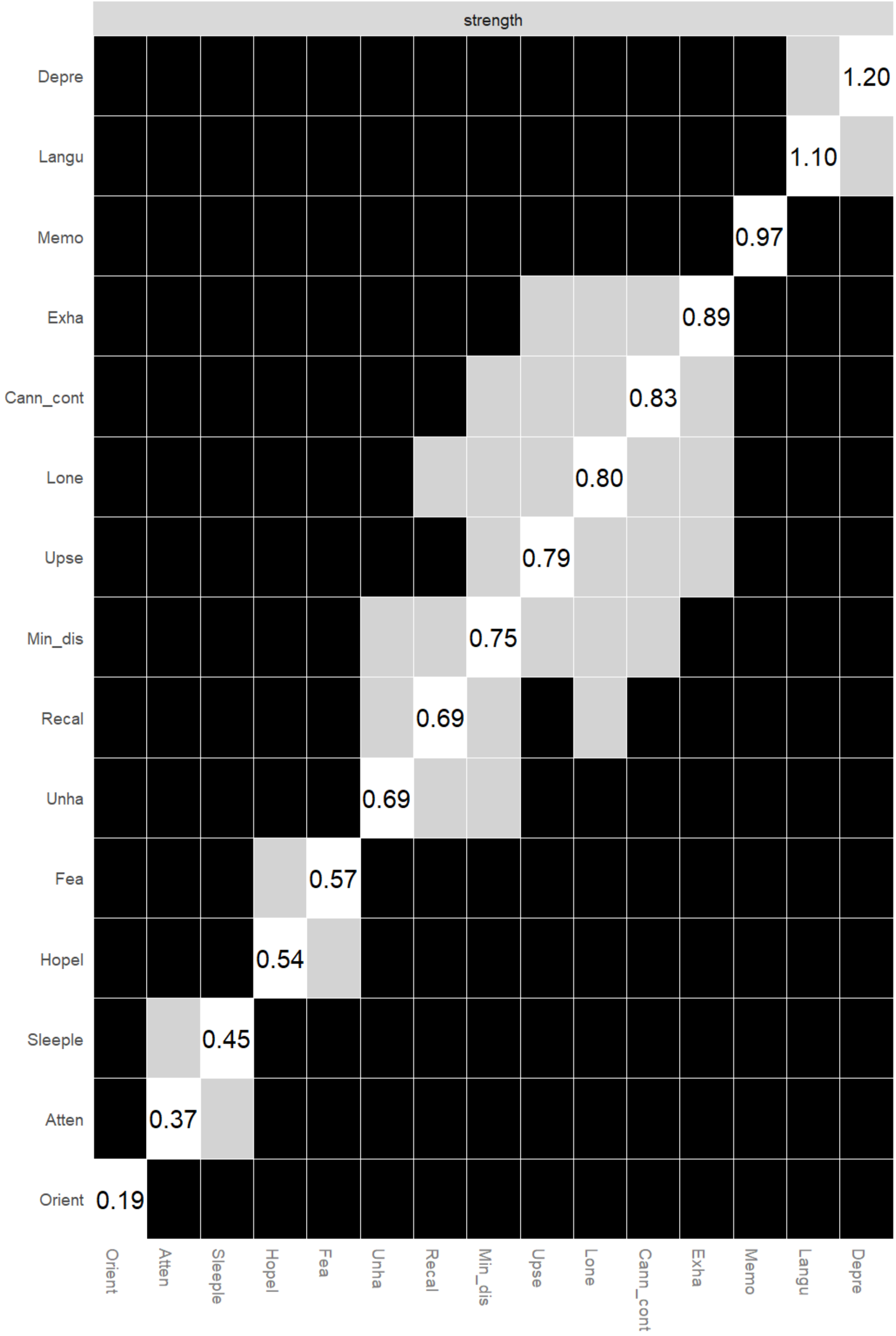

Non-grandparenting

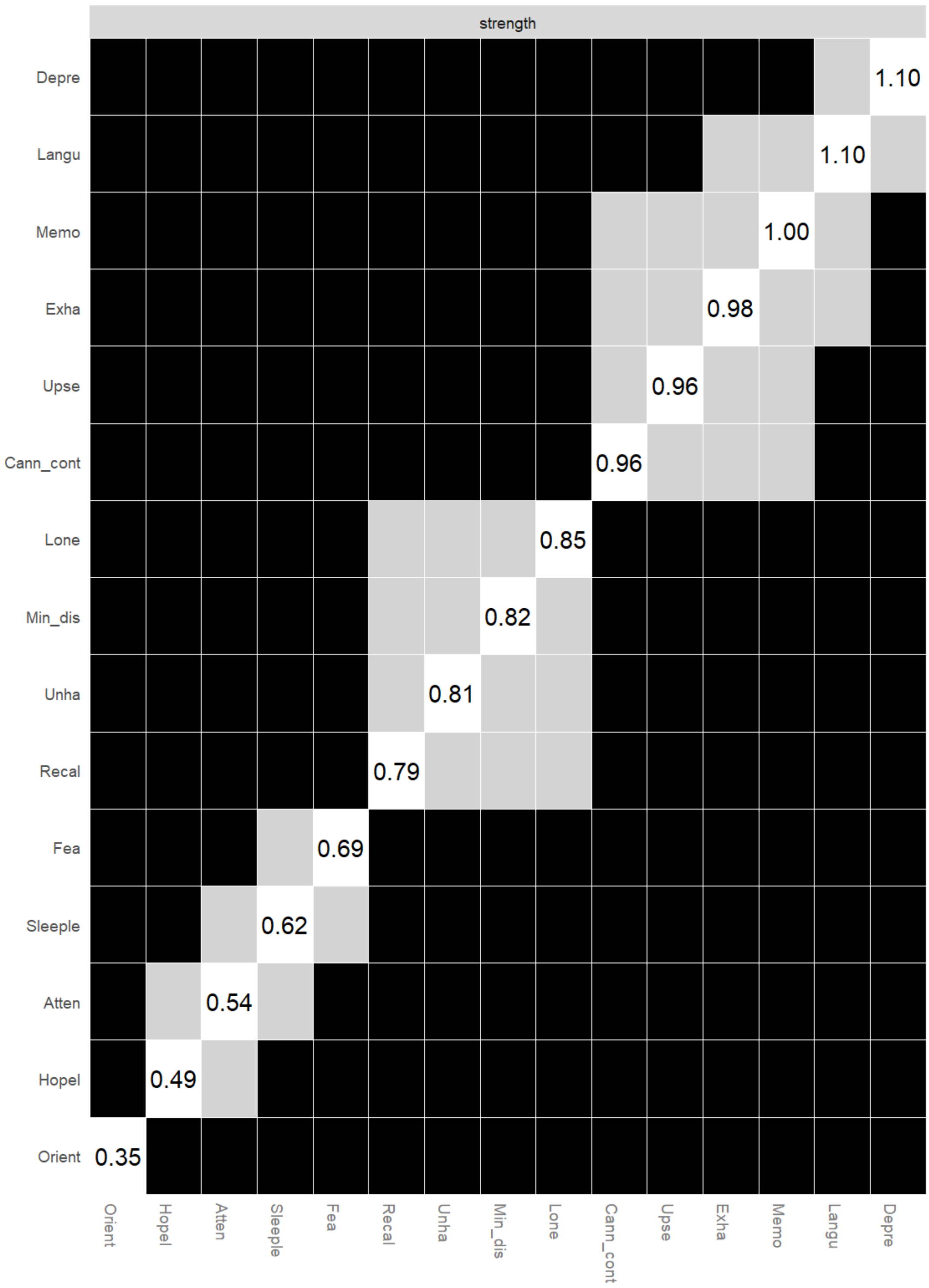

Appendix S6. **Accuracy of the edge-weights for the current network model.** The gray area represents the 95% Confidence Intervals of edge weights, estimated with the non-parametric bootstrap procedure. Wide intervals indicate lower stability and narrow intervals indicate higher stability. The red dots indicate the sample values, while the black dots indicate the values of each edge weight, ordered from the highest to the lowest value.

Grandparenting

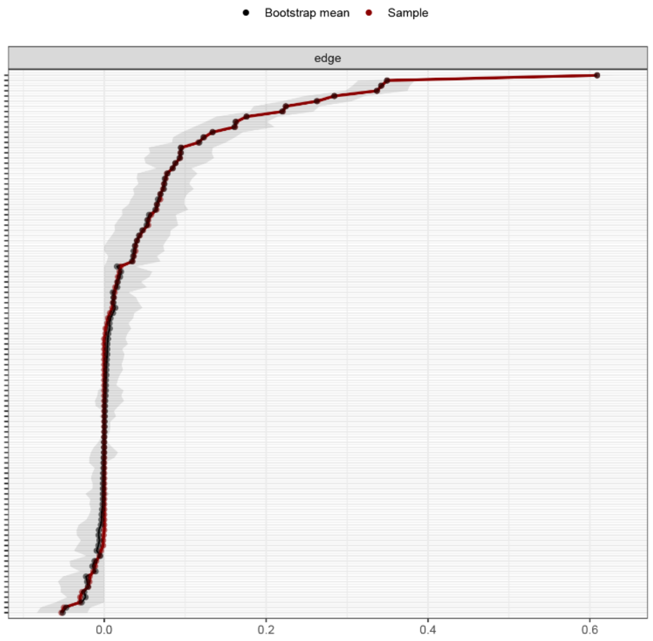

Non-grandparenting

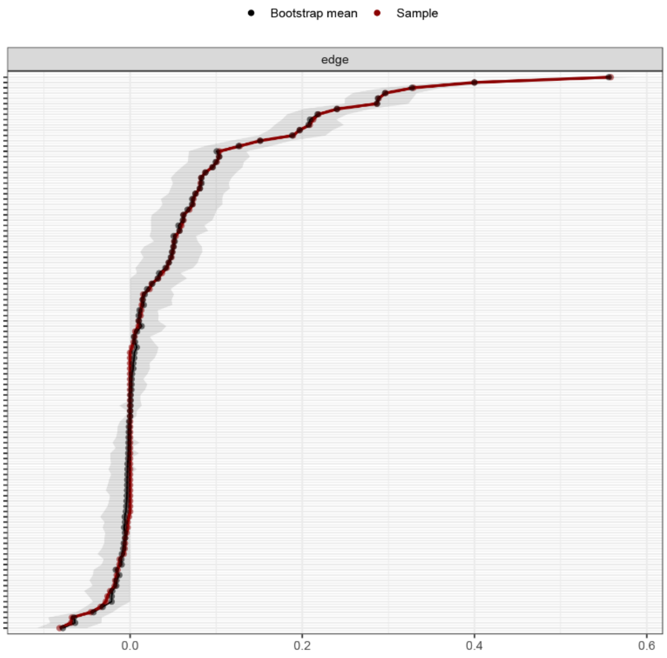

Appendix S7 Estimation of edge weight difference by bootstrapped difference test. Bootstrapped difference tests between edge weights in the network. Gray boxes indicate edges that do not significantly differ from one another. Black boxes represent edges with a significant difference from one another (α = 0.05). Blue boxes in the edge-weight plot indicate positive correlations, and orange boxes in the edge-weight plot indicate negative correlations

Grandparenting

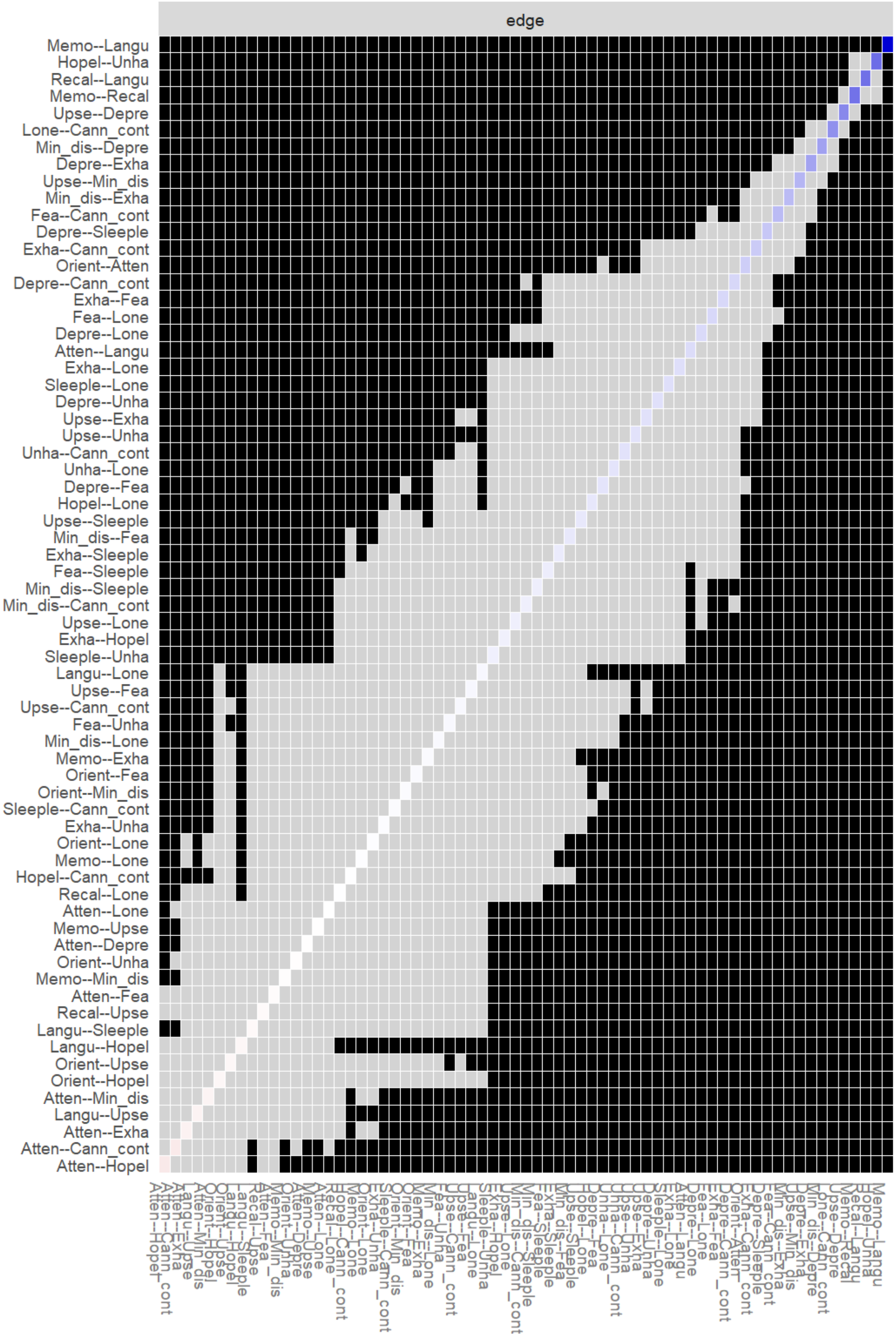

Non-grandparenting

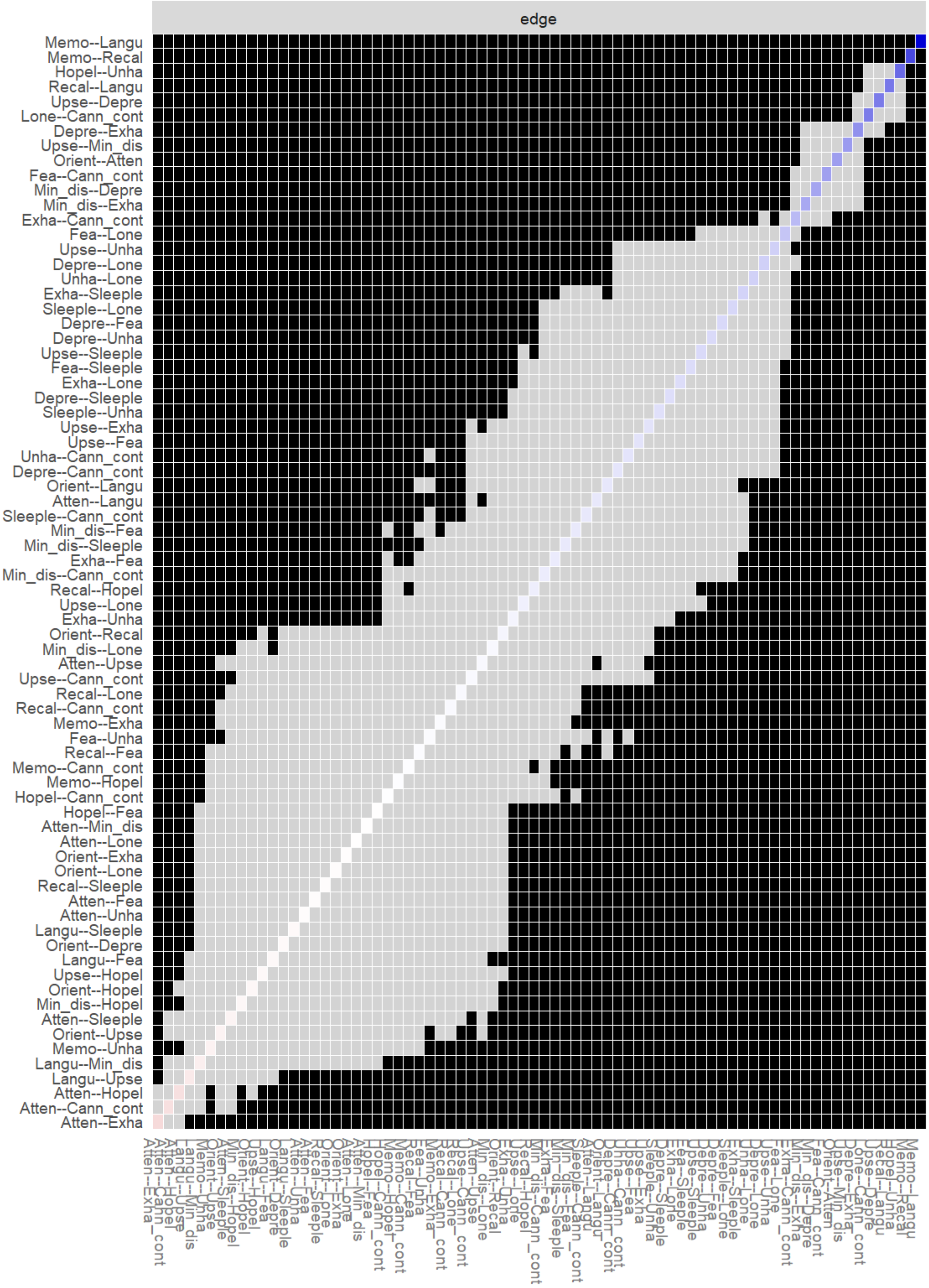

Appendix S8 Stability of centrality indices by case dropping subset bootstrap. The x-axis represents the percentage of cases of original sample used at each step. The y axis represents the average of correlations between the centrality indices from the original network and the centrality indices from the networks that were re-estimated after dropping increasing percentages of cases. Each line indicates the correlations of betweenness, strength and closeness, while areas indicate 95% CI.

Grandparenting

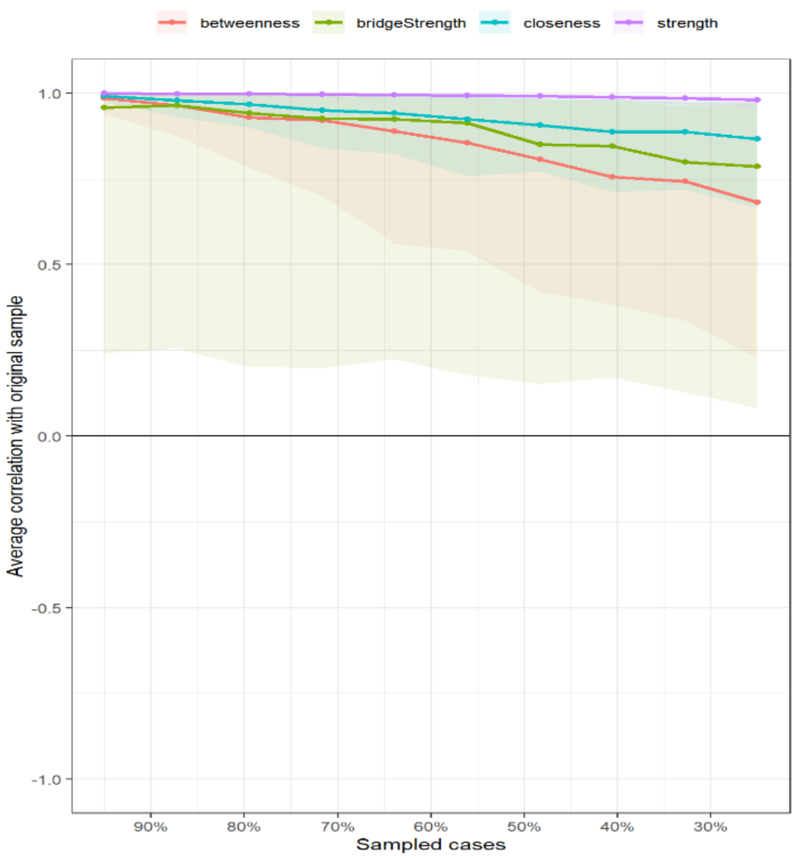

Maximum drop proportions to retain correlation of 0.7 in at least 95% of the samples:

betweenness: 0.283

- For more accuracy, run bootnet(..., caseMin = 0.206, caseMax = 0.361)

bridgeStrength: 0.283

- For more accuracy, run bootnet(..., caseMin = 0.206, caseMax = 0.361)

closeness: 0.75 (CS-coefficient is highest level tested)

- For more accuracy, run bootnet(..., caseMin = 0.672, caseMax = 1)

strength: 0.75 (CS-coefficient is highest level tested)

- For more accuracy, run bootnet(..., caseMin = 0.672, caseMax = 1)

Non-grandparenting

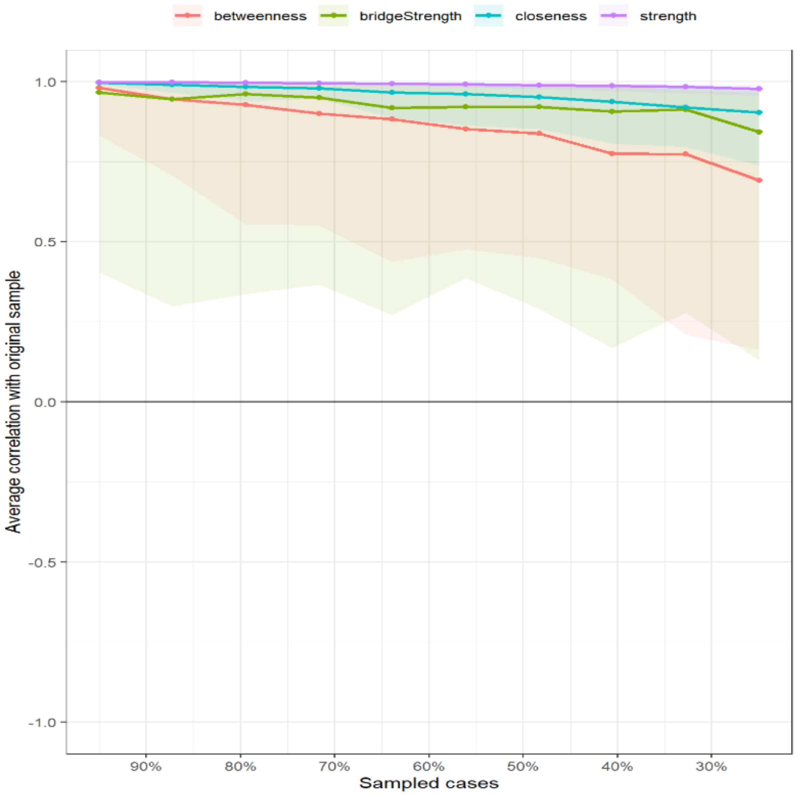

Maximum drop proportions to retain correlation of 0.7 in at least 95% of the samples:

betweenness: 0.206

-For more accuracy, run bootnet(..., caseMin = 0.128, caseMax = 0.283)

bridgeStrength: 0.206

-For more accuracy, run bootnet(..., caseMin = 0.128, caseMax = 0.283)

closeness: 0.75 (CS-coefficient is highest level tested)

-For more accuracy, run bootnet(..., caseMin = 0.672, caseMax = 1)

strength: 0.75 (CS-coefficient is highest level tested)

-For more accuracy, run bootnet(..., caseMin = 0.672, caseMax = 1)
